# Novel mechanisms, treatments, and outcome measures in childhood sleep

**DOI:** 10.3389/fpsyg.2015.00602

**Published:** 2015-05-12

**Authors:** Annalisa Colonna, Anna B. Smith, Deb K. Pal, Paul Gringras

**Affiliations:** ^1^Department of Basic and Clinical Neurosciences, Institute of Psychiatry, Psychology and Neuroscience, King’s College London, London, UK; ^2^Paediatric Neurosciences Unit, Evelina London Children’s Hospital, St. Thomas’ Hospital, London, UK

**Keywords:** sleep, pediatric cognition, learning, memory, outcome measure, sleep treatments

## Abstract

Sleep disorders and sleep of insufficient duration and quality are on the increase due to changes in our lifestyle, particularly in children and adolescents. Sleep disruption is also more common in children with medical conditions, compounding their difficulties. Recent studies have focused on new mechanisms that explain how learning and cognitive performance depend on a good night’s sleep. Growing alongside this latest understanding is an innovative new field of non-drug interventions that improve sleep architecture, with resulting cognitive improvements. However, we need to rigorously evaluate such potentially popular and self-administered sleep interventions with equally state-of-the-art outcome measurement tools. Animated hand-held games, that incorporate embedded sleep-dependent learning tasks, promise to offer new robust methods of measuring changes in overnight learning. Portable computing technology has the potential to offer practical, inexpensive and reliable tools to indirectly assess the quality of sleep. They may be adopted in both clinical and educational settings, providing a unique way of monitoring the effect of sleep disruption on learning, leading also to a radical rethink of how we manage chronic diseases.

We are witnessing an explosion of original and exciting research in sleep medicine that will transform our understanding of learning, memory, and behavior in children. There are three key domains that are advancing with astonishing speed and have exciting potential synergies. First, there are new understandings of the mechanisms that link sleep quantity and quality with daytime cognitive and behavioral performance. Second, is the invention of novel non-medication based interventions that improve sleep architecture with resulting cognitive improvements; and third is the emerging interface between tablet-based multimedia gaming and psychometrics.

Sleep is now understood as an active process, crucial for organizing and selectively retaining salient knowledge (Stickgold and Walker, 2013). Fragile temporary memory traces are transformed and consolidated during sleep into stable and persistent traces within our long-term memory (Stickgold and Walker, 2013; Diekelmann, 2014). Different sleep stages during the night all seem to have discrete roles and selectively reinforce specific types of learning: research in adults demonstrates the role of slow-wave sleep in consolidating learning about facts and knowledge, while rapid eye movement sleep, the “dream state,” is more important for consolidating motor skills (Rasch and Born, 2013). In children by contrast, the consolidation of learning about facts and knowledge may well take priority over the learning of new motor skills in sleep. Children have both stronger slow-oscillations and more slow-wave sleep than adults, and this might explain the differential effect (Wilhelm et al., 2012).

About 40% of children experience sleep problems at some point during their development (Alfano and Gamble, 2009). Well described secular changes like early school start times, more homework, and busy evenings on social media using light-producing (but melatonin inhibiting) electronic devices can all interfere with sleep patterns (Alfano and Gamble, 2009). It seems likely that the resulting decline in total sleep duration of children and adolescents is linked to these changes in behavior. In particular, the use of electronic media at night may well interfere with sleep quality and quantity (Cain and Gradisar, 2010). Sleep problems are seen in over half of all children with neurodevelopmental problems, such as intellectual disability, epilepsy, autism spectrum disorders, and attention deficit hyperactivity disorder; many physical conditions including intrinsic sleep disorders, asthma, allergies, gastro-esophageal reflux, and pain also reduce sleep (Alfano and Gamble, 2009; Sadeh and Sivan, 2009; Wilhelm et al., 2012). These environmental and clinical disturbances have been associated with disrupted sleep as well as impaired daytime cognitive and behavioral performance (Kheirandish and Gozal, 2006; Alfano and Gamble, 2009; Wilhelm et al., 2012).

Treatment of some of the more subtle deficits in sleep quality and quantity are difficult: sleep medications for children have higher rates of adverse effects, hangover effects and residual cognitive impairments than in adults. However, a handful of new non-medication strategies offer an exciting route forward. The first strategy is the appealingly simple 30 min of hard exercise, 3 or 4 h prior to sleep: a small but well-controlled study demonstrated vigorous afternoon exercise improves sleep efficiency and percentage of slow-wave sleep in children (Dworak et al., 2008). Second on the list is the potential for a “deep-sleep specific” hypnotic suggestion to improve sleep. This strategy, using metaphorical sentences that symbolize the depth of sleep, spoken in a calm and relaxed manner, increased slow-wave sleep in a nap-study of adults, who are generally less susceptible to hypnotic suggestion than children (Cordi et al., 2014). The next two innovations involve timing an external intervention to coincide with slow-wave sleep in the early part of the night. One uses transcranial oscillating direct current via electrodes either side of the forehead. This painless technique led to remarkably enhanced slow oscillation power and elevated memory consolidation in some tasks to the level of healthy controls in a recent study of 12 boys with attention deficit hyperactivity disorder (Prehn-Kristensen et al., 2014). Another technique uses auditory stimulation with bursts of pink noise (computer generated noise that sounds like rainfall) cleverly generated to rhythmically coincide with specific parts of the subject’s intrinsic slow oscillations. This, like electrical stimulation, seemed to boost slow oscillations, spindles (another aspect of sleep architecture believed to be crucial for learning), and recall of word pairs in adults (Ngo et al., 2013).

The enhancement of memory during sleep is likely to become a very exciting new field, promising to change our future research and to lead to new practical applications. We acknowledge that there are ethical dilemmas to be considered, such as trade-off effects (enhancing one memory at the cost of impairing others; Payne and Kensinger, 2011) or potential rebound mechanisms whereby sleep enhancement on one night may be followed on the following night by corrective curtailment of sleep processes. A major consideration should also be our vulnerability during sleep and our protection during any interventions while we are in this state (Diekelmann, 2014).

The evaluation of the above innovative interventions will require equally state-of-the-art outcome measurement tools. The effectiveness of these sleep interventions needs to be measured through a new outcome measure not only in terms of improvement in sleep architecture, but also in terms of cognitive change.

The use of polysomnography, a nocturnal continuous electrophysiological recording of brain activation, eye movements, skeletal muscle activation, and heart rate, represents a gold standard investigation to assess sleep architecture (Markovich et al., 2014), but is time-consuming, intrusive and costly. Actigraphy, measured with wrist-watch like devices, provides a convenient alternative that is able to estimate sleep efficiency by recording gross motor activity in a valid, ecological and economical way (Markovich et al., 2014). While neither of these approaches can inform us of cognitive changes that occur during sleep, they could be partnered with equally ecological, valid and practical tools designed to measure cognition and memory changes.

New tools which involve testing declarative and procedural memory skills will allow us to measure the change in memory consolidation between daytime and overnight intervals. Experimenters have employed a variety of paradigms [e.g., finger sequence tapping, serial reaction-time task, word-pair learning paradigms, lexical integration of novel words, and emotional memory tasks (Rasch and Born, 2013)] in carefully controlled conditions. But these tests need direct administration, and they are often not appealing to children, making it difficult to translate findings from such paradigms into real life learning scenarios. We know children appear not to retain all the information they are exposed to during the day; instead, information is selected in the form of a “memory triage” based upon salience (Stickgold and Walker, 2013). Therefore a major concern is that learning through unimaginative, artificial paradigms lacking salience may not be well consolidated.

There are specific memory processes that are well established as sleep sensitive and which seem ideal to capture in a set of outcome measures: both spatial learning (Nguyen et al., 2013) and target detection, specifically where stimuli have an emotional loading (Soffer-Dudek et al., 2011), have been shown to be sensitive to sleep. We feel that any potential measure of sleep-related learning should also focus upon language consolidation, since language development is crucial for future learning and any enhancement of this skill has “real world” rather than theoretical meaning; we also know that the integration of new vocabulary into existing semantic networks takes place during sleep (Tamminen et al., 2013); and finally, in several neuropediatric disorders (e.g., epileptic encephalopathy) disrupted sleep is often coupled with language impairment. Examples of tasks measuring these three domains of sleep enhanced learning are illustrated in Figure 1.

**FIGURE 1:**
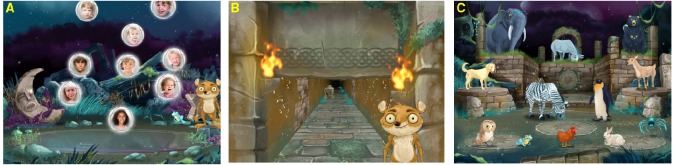
**Three paradigms suitable for an outcome measure for sleep dependent learning. (A)** Target detection of emotional stimuli: a continuous performance task which uses children’s faces as stimuli. Targets are smiling expressions and non-targets are neutral or sad expressions of emotion. Performance is evaluated through reaction time, intra-individual standard deviation of reaction time, commission and omission errors. **(B)** Spatial learning: a 2D map is provided briefly for a 3D maze learning task. Performance is evaluated through number of decision points passed; total decision time; and navigation speed (number of decision points/total decision time). **(C)** Language learning: a set of animals is presented together with their names in an unfamiliar language. After a 12 min duration the unfamiliar animal names have to be recalled. Performance is evaluated through number of animals correctly recalled.

These new tools should show high retest reliability, should be scalable and affordable; user-friendly and engaging for children; and they should be autonomous, rather than requiring tester supervision, presented on an easily portable and user-friendly tablet device. This set of challenges naturally leads us to consider lessons from the field of gaming, where the issues of motivation, sustaining attention, consistency, accessibility, and scalability are inherently part of the design brief.

A marriage of technology and neuroscience may help us to adapt this ready-made technology to meet robust psychometric principles. Moreover, utilizing gaming expertise can help us address issues such as task development, standardization, and the collection of normative data, with the aim of producing child friendly games that allow us to measure memory consolidation. As illustrated in Figure 2, the first step will be to establish these outcome measures as valid indices of memory consolidation by correlating them with gold standard polysomnography measures to show that improved performance is associated with physiological changes in sleep architecture. Once validated, these games can advance our understanding of how environmental and clinical conditions impact sleep and learning, with potential implications for the way chronic conditions are managed. We will also be able to monitor and evaluate the effect of medications such as stimulants, selective serotonin reuptake inhibitors and some antiepileptic drugs, such as benzodiazepines and barbiturates, which may increase sleep disruption in some children (Alfano and Gamble, 2009; Chan et al., 2011). We hope that, with a nudge in the right direction, translational tools for measuring, evaluating and monitoring sleep-related learning and interventions could be delivered, in the near future, on an iPad near you.

**FIGURE 2:**
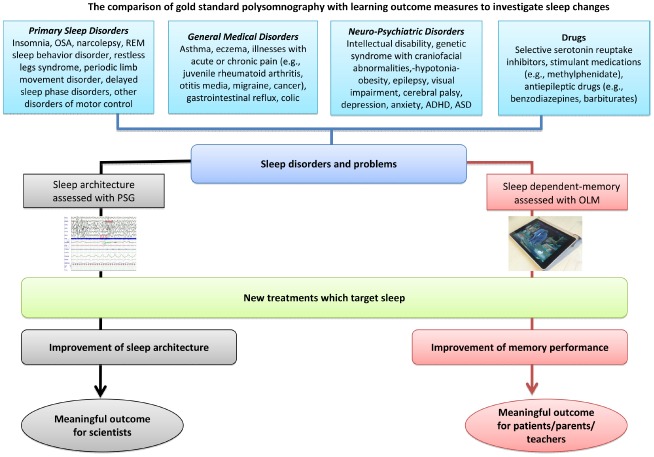
**Routes for assessing the effect of intervention in sleep disruption.** OLM, overnight learning measure; OSA, obstructive sleep apnea; PSG, polysomnography; REM, rapid eye movement.

## Author Contributions

AC: substantial contributor to the conception of the work, first draft and final approval of the version to be published. AS: substantial contributor to the design of the work, revising it critically for important intellectual content and final approval of the version to be published. DP: substantial contributor to the conception of the work, revising draft critically and final approval of the version to be published. PG: substantial contributor to the conception of the work, revising it critically for important intellectual content and final approval of the version to be published.

### Conflict of Interest Statement

The authors declare that the research was conducted in the absence of any commercial or financial relationships that could be construed as a potential conflict of interest.
